# Size Dependence of the Magnetoelastic Properties of Metallic Glasses for Actuation Applications

**DOI:** 10.3390/s19194296

**Published:** 2019-10-04

**Authors:** Ariane Sagasti, Jon Gutiérrez, Andoni Lasheras, José Manuel Barandiarán

**Affiliations:** 1 Department of Electricity and Electronics, Universidad del País Vasco/Euskal Herriko Unibertsitatea (UPV/EHU), Barrio Sarriena s/n, 48940 Leioa, Spain; ariane.sagastis@ehu.eus (A.S.); josemanuel.barandiaran@ehu.eus (J.M.B.); 2 BCMaterials, Basque Center for Materials, Applications and Nanostructures, UPV/EHU Science Park, Barrio Sarriena s/n, 48940 Leioa, Spain; 3 Department of Applied Physics I, Universidad del País Vasco/Euskal Herriko Unibertsitatea, Engineering School, 48005 Bilbao, Spain; andoni.lasheras@ehu.eus

**Keywords:** magnetoelasticity, *ΔE* effect, Young’s modulus, magnetoelastic coupling, resonance quality factor

## Abstract

We present an exhaustive study of the magnetoelastic properties of 24 strips with different rectangular dimensions, cut from a long ribbon of Metglas^®^ 2826MB3. The strips have a length-to-width ratio *R = L/w* ranging from 2 to over 20. Significant variations of the apparent saturation Young’s modulus and the *ΔE* effect with strip geometry, changing from 160 GPa and 4% for *L* = 10 mm, *w* = 5 mm and *R* = 2, to 164 GPa and 9.6% for *L* = 35 mm, *w* = 1.7 mm and *R* = 20.6, have been observed. In order to obtain the highest values of the *ΔE* effect, the magnetomechanical coupling coefficient, *k*, and the quality factor of the resonance, *Q*, a value *R* > 14 is needed. The effective anisotropy field H_k_^*^, taken as the minimum of the *E(H)* curve, and its width *ΔH*, are not as strongly influenced by the *R* value, and a value of *R* > 7 is enough to reach the lowest value. From our measurements we infer that the formerly predicted value of *R* > 5 needed for a good magnetic and magnetoelastic response of the material must be actually regarded as the lowest limit for this parameter. In fact, we show that the demagnetizing factor *N*, rather than the length-to-width ratio *R*, is the parameter that governs the magnetoelastic performance of these strips.

## 1. Introduction

Metallic glasses working as magnetoelastic resonators show excellent magnetomechanical behavior due to their good values of spontaneous magnetization and saturation magnetostriction, and low magnetocrystalline anisotropy [1,2,3,4]. These properties arise during their fabrication process by ultra-fast cooling after the melt, a procedure that makes the single-roller quenching method the most used one and yields a metallic glass product fabricated in the form of long ribbons.

**Figure 1:**
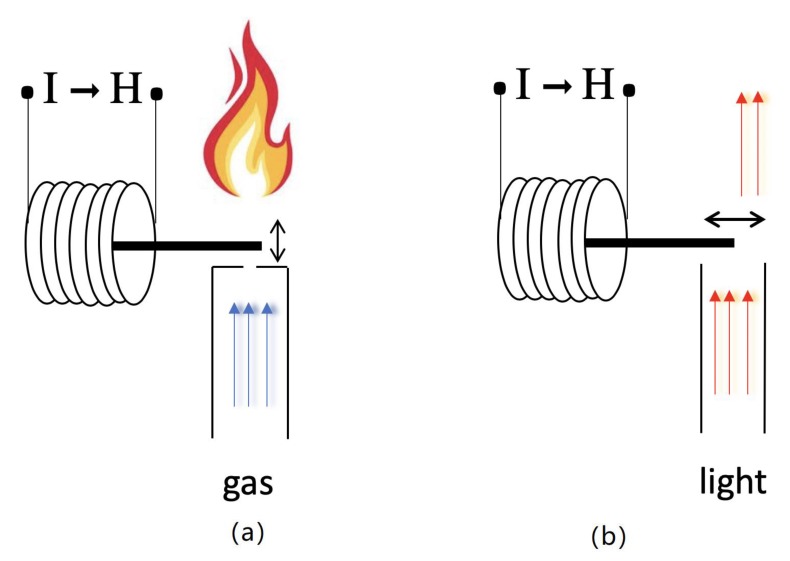
A schematic proposal for: (**a**) a simple open/close gas valve, or (**b**) light flux shutter controller.

When working as sensing magnetic materials, the magnetoelastic resonance frequency of a metallic glass element is extremely sensitive to any mass added to the resonator [5,6]. This configuration allows remote query and answer [7], as well as low manufacturing cost and low power consumption [8] for the interrogation. For this reason, they have been used to detect a wide range of environmental, physical, chemical and biological parameters (see, for example, references in [9,10]). Nevertheless, little work has been done using such materials as actuators. The possibility to control metallic glass strip elongation by using an externally applied or bias magnetic field opens new application possibilities as a simple two-state open/close gas valve [11] or an extremely precise light flux shutter controller (see Figure 1).

There is a clear influence of the geometric shape of amorphous ferromagnetic materials in the observed magnetic and magnetoelastic behavior. In particular, for rectangular ribbon-shaped strips of metallic glasses, several authors [12,13,14] have already demonstrated that a value of *R* > 5, *R* being the length-to-width ratio, is needed for a good magnetic and magnetoelastic response of the material. In many practical applications this condition is rarely applied.

In order to gain a better understanding of the dependence of magnetoelastic properties on resonator geometry, we performed an exhaustive study of the main parameters involved in the resonance: change *E(H)* of the apparent Young modulus with the applied field, *ΔE* effect, maximum magnetoelastic coupling coefficient *k_max_*, and corresponding quality factor of the resonance, *Q*. For this study, strips of different length-to-width ratio *R = L/w* (ranging from 2 to 20.6) were cut from a single long ribbon of Metglas^®^ 2826MB3 to ensure they shared the same basic properties. The results indicate large differences in the measured main magnetoelastic parameters, differences that will be qualitatively analyzed as a function of the length-to-width ratio *R* value of each strip.

## 2. Materials and Methods

Metglas^®^ 2826MB3 (Fe_37_Ni_42_Mo_4_B_17_, see [15]), was chosen for the experiments. It is a commercial material often used in magnetoelastic sensor applications due to its good magnetic and magnetoelastic properties, and resistance to corrosion [16,17]. Room-temperature hysteresis loops of the material were measured by a classical induction method, obtaining a saturation magnetization *μ_0_M_S_* = 0.88 T and initial susceptibility χ = 15,000. Saturation magnetostriction was determined by using strain gages connected to an electronically balanced bridge, obtaining a value of *λ_S_* = 12 ppm.

Starting from a long ribbon of Metglas^®^ 2826MB3 of 30 µm thickness in as-cast state, strips were laser cut with perfect rectangular shape of length varying from *L* = 35 mm to *L* = 10 mm in steps of 5 mm, and in four different widths: *w* = 5.0 mm, 3.33 mm, 2.5 mm and 1.66 mm. That is, the initial value of *w* = 5 mm of the long ribbon was reduced in a factor of 2/3, 1/2 and 1/3, respectively. Following this procedure, we obtained a set of 24 different rectangular strips and subsequently a set of magnetoelastic resonators with different length-to-width ratios *R = L/w*, ranging from 2 to 20.6.

We used a home-mounted magnetoelastic resonance detection apparatus to measure the magnetoelastic resonant (*f_r_*) and anti-resonant (*f_a_*) frequencies, at an external applied magnetic field (or bias) *H*. Precise determination of frequencies was performed using a Hewlett Packard 3589A Spectrum Analyzer, working in the 50–250 kHz range. A detailed description of the set-up can be found in [9,18]. Due to magnetoelasticity, the measured resonant frequency (*f_r_*) will vary with the bias field *H*, and so too will the Young’s modulus, determined as $E\left(H\right)={\left[2Lf_{r}\left(H\right)\right]}^{2}\rho$ [19], where *L* and *ρ* are the length and density of the sample. This field-dependence of the elastic modulus is known as *ΔE* effect and is usually given in % variations:$\;\Delta E\;\left(\%\right)=(1-E\left(H\right)/E_{S})\times 100$, *E_S_* being the Young’s modulus measured at magnetic saturation. Other important magnetoelastic parameters that can be determined from these measurements are the magnetomechanical coupling coefficient ($k^{2}\;=\;\left(\pi^{2}/8\right)\left(1-\;{\left(f_{r}/f_{a}\right)}^{2}\right)$) [20] and quality factor of the resonance $\left(Q=f_{r}/\Delta f\right)$, all quantities being function of the applied external magnetic field.

Finally, for some applications (such as electronic article surveillance [21]) high-anisotropy fields combined with a wide *ΔE* effect curve are necessary. Consequently we have also followed the behavior of the effective anisotropy field $H_{k}^{*}$ [14] or bias field necessary to reach the minimum of the *E(H)* value (corresponding to the maximum value of the *ΔE* effect) and the width *ΔH* of this *E(H)* curve at half of its maximum depth.

## 3. Results

**Figure 2:**
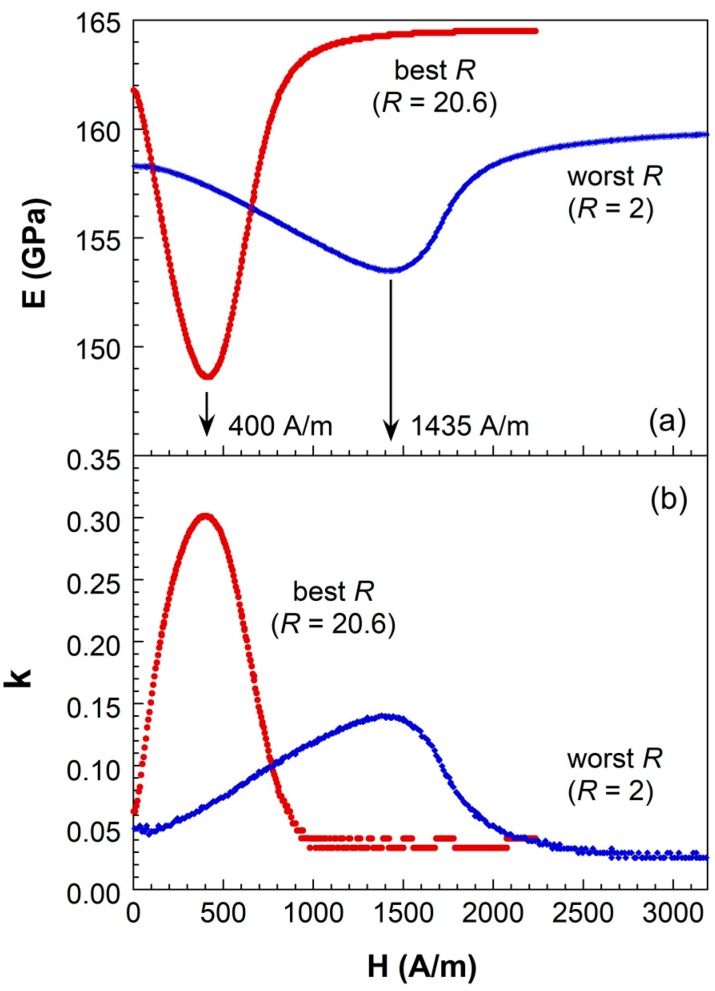
(**a**) *ΔE* effect and (**b**) magnetoelastic coupling coefficient measured for strips of Metglas^®^ 2826MB3 with the highest (*R* = 20.6) and the lowest (*R* = 2) *R* value.

The drastic influence of the geometric shape (as indicated by the *R* factor) on the magnetoelastic behavior of the studied strips is clearly observed in Figure 2, where *E(H)* and *k(H)* bias field dependence, Young’s modulus and magnetoelastic coupling coefficient respectively, are shown for the highest (best) and lowest (worst) *R* values. 

The magnetoelastic behavior of the strip with the lowest *R* (corresponding to *L* = 10 mm, *w* = 5 mm) value is worse than that measured for the strip with the highest *R* (corresponding to *L* = 35 mm, *w* = 1.66 mm). The saturation Young’s modulus value *E_S_* and *ΔE* effect magnitude decrease from 164.5 GPa and 9.6% to 159.9 GPa and 4%. Similarly, maximum magnetoelastic coupling reduces from 0.3 to 0.14. All these changes are accompanied by an evident increase in the bias external field necessary to reach those minima in the *E(H)* and maxima in the *k(H)* behaviors, from 400 to 1435 A/m. 

**Figure 3:**
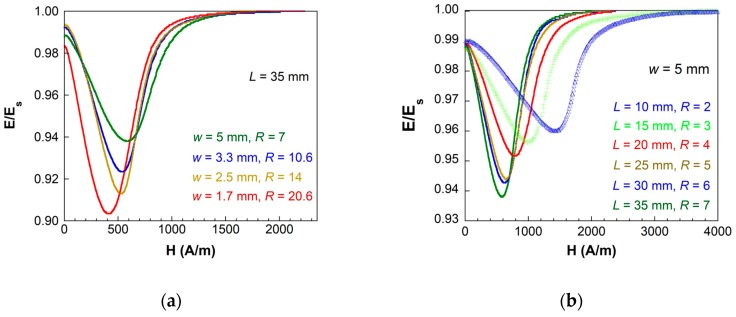
*ΔE* effect measured with: (**a**) *L* constant and variable *w* and (**b**) *w* constant and variable *L*.

Considering all the lengths and widths of the strips used in this study, Figure 3 shows the continuous change observed in the measured *E(H)* curves when keeping the length *L* of the strips constant (*L* = 35 mm, Figure 3a) and when keeping the width *w* of the strips constant (*w* = 5 mm, Figure 3b). Curves for different lengths *L* and different widths *w* of the strips behave in a similar way, that is to change the R ratio by changing *L* or *w*, influencing the *ΔE* effect magnitude, the applied bias external field necessary to reach the minima, and also the width of the *E(H)* curve.

All the magnetoelastic parameters estimated from the measured *E(H)* and resonance/anti-resonance frequency measurements performed for all the studied Metglas^®^ 2826MB3 strips with different lengths *L* and different widths *w* are summarized in Table 1.

In the following we will plot and analyze the behavior of these quantities: *ΔE* effect, effective anisotropy field $H_{k}^{*}$, maximum magnetoelastic coupling *k_max_*, the quality factor of the corresponding resonance, *Q*(*k_max_*), and the width *ΔH* of the measured *E(H)* curves, as a function of the length-to-width ratio *R*.

**Figure 4:**
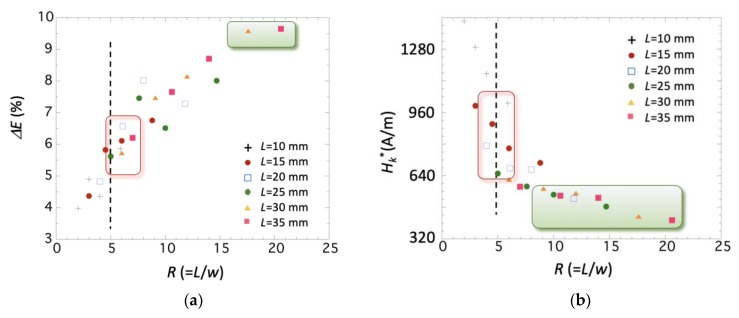
Measured (**a**) *ΔE*(%) magnitude and (**b**) $H_{k}^{*}$ value as a function of the length-to-width ratio *R*, for all the measured strips.

**Figure 5:**
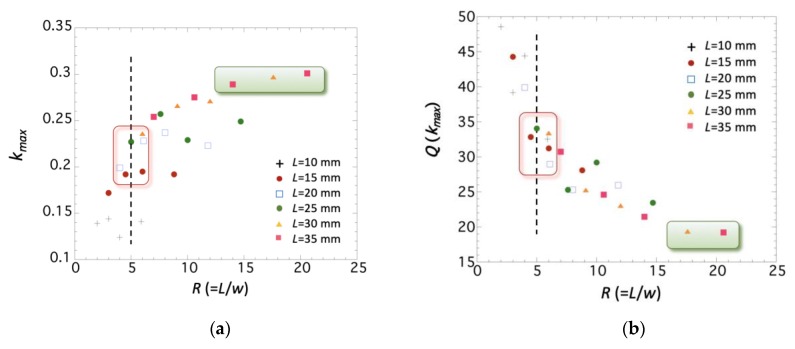
Measured (**a**) *k_max_* value and (**b**) *Q*(*k_max_*) value as a function of the length-to-width ratio *R*, for all the measured strips.

Figure 4 and Figure 5 show that all these parameters have a strong dependence on the value of *R*. The condition *R* ≥ 5 is insufficient to guarantee a good magnetoelastic behavior: the *ΔE* effect reaches its maximum value (about 10%) at *R* > 15, being almost 67% higher than the measured one at *R* = 5 (about 6–7%). In an analogous way, the maximum magnetoelastic coupling *k_max_* reaches its maximum value (about 0.3) at *R* > 12, while at *R* = 5, it remains at about 0.2–0.25. Corresponding *Q*(*k_max_*) at those same *R* values are the lowest measured, with a value about 20, while strips with value of *R* = 5 had resonance quality factors ranging from about 30 to 35.

**Figure 6:**
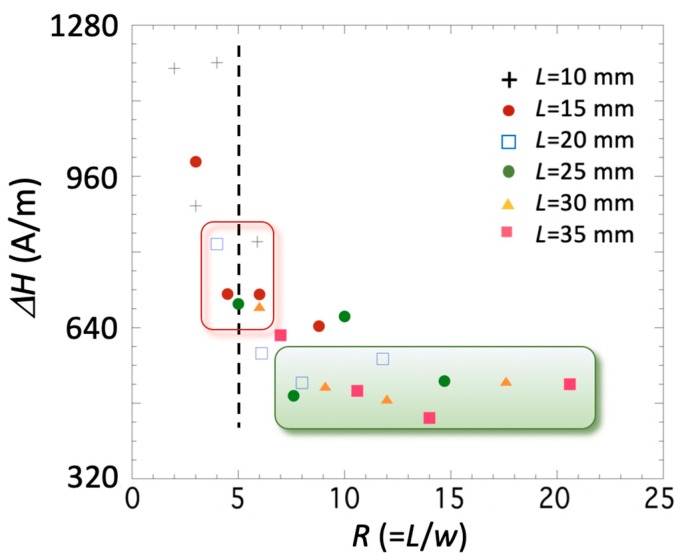
Measured *ΔH* width as a function of the length-to-width ratio *R* for all the measured strips.

The parameters that are less influenced by the change in the length-to-width ratio *R* are the effective anisotropy field $H_{k}^{*}$ (see Figure 4b) and the width *ΔH* of the measured *E(H)* curves (see Figure 6). This effective anisotropy field $H_{k}^{*}$ is, in our case, intrinsic to the material and arises from its fabrication process, so any observed change must arise from the different shapes (*R* values) studied.

In both cases the observed behavior is analogous, that is, the field $H_{k}^{*}$ and the width *ΔH* decrease as the *R* value increases. Nevertheless—and despite the previously described behavior of the magnetoelastic parameters—it is enough to use strips with *R* ≥ 7–8 in order to obtain the lowest effective anisotropy field (about 400–560 A/m) and width *ΔH* (about 440–560 A/m). 

## 4. Discussion

It is already well-established that the dimensions of ribbon-shaped magnetoelastic materials affect their magnetomechanical response—that is, the resulting shape anisotropy for resonant platforms of different aspect ratios reflects different demagnetizing factors that can severely reduce the observed magnetomechanical coupling and related properties. The demagnetizing factor *N* relates the *H* bias field applied to our magnetoelastic specimen, its magnetization *M* and the internal effective field *H_eff_* through the expression:

(1) $$H_{eff}=H-N\cdot M$$

The quantity “−H·M” is known as the internal demagnetizing field. Strictly speaking, unless the specimen has a revolution ellipsoidal shape, this demagnetizing field will be non-uniform along the strip length. This observation in the case of soft amorphous magnetic microwires (of cylindrical symmetry) has been extensively reported by Usov [22] and Zhukova et al. [23]. In the following example, intended to give an adequate interpretation of our experimental results, let us assume the demagnetizing factor *N* to be constant through the section of our ribbons. The corresponding value (for each *R* value case) will be taken from the extensive previous works performed by Chen [24,25] in which a deep reasoning about the variation of the demagnetizing factor *N* of rectangular prisms together with its calculation is reported.

From Equation (1) it is inferred that the higher the demagnetizing effect, the lower the measured susceptibility $\chi_{eff}$ [26] given as:

(2) $$\chi_{eff}=\frac{\chi }{1+N\cdot \chi }$$

where χ is the true or intrinsic susceptibility of the material. Similarly, this will happen with the magnetoelastic properties of the ribbon [27] like the *ΔE* effect, that will reduce in the form:

(3) $$\Delta E_{eff}=\frac{1}{1+N\cdot \chi }\Delta E$$

The quantity 1/(1+N·χ) has been already defined as a “reduction factor, *RF*” that affects any property concerning magnetostrictive/magnetoelastic constituents, e.g., the magnetoelectric effect observed in laminated PVDF/Metglas composites [14].

So, the measured magnitude of the (magnetoelastic) properties of these type of materials will be close to the true value (e.g., $\Delta E_{eff}\approx \;\Delta E$) when the demagnetizing factor *N* → 0, that is, when the ribbon-shaped magnetoelastic specimen has a large length-to-width aspect ratio *R.* Following the works by Schmidt et al. [12,13], a length-to-width aspect ratio of 5:1 was considered enough to guarantee accurate magnetoelastic resonance measurements. They justified their argument in Figure 9 of their work [12], in which, for *R* > 5 values, a constant velocity of sound (about 4400 m/s) in Metglas^®^ 2826MB3 was found, so a uniform applied bias *H* field along the longitudinal axis of the ribbon was assumed. 

As we will show in the following, *R* and *N* do not have direct proportionality, and even in the case of *R* > 5, where a constant sound velocity through the strips is achieved [12,13], other magnetoelastic parameters can still vary. This can be clearly seen in Table 2, where strips with values of *R* equal or close to 6, but with different lengths, have been extracted from Table 1 and compared. 

It can be observed that the random and small changes in *E_min_*, *Es* and *ΔE* effect (about 1% for all of them) is accompanied by a monotonous decrease of $H_{k}^{*}$ and *ΔH*, and also an increase in the magnetoelastic coupling, *k_max_*, mainly due to the different *L* and *w* values.

In fact, and following previous works performed by Chen et al. [24,25], which report variation in the demagnetizing factor *N* with χ and the aspect ratio of the sample, we can check by extrapolation the fluxmetric demagnetizing factor *N_f_* of a general rectangular prism of dimensions 2*a* × 2*b* × 2*c* along *x*, *y*, *z* axis respectively, so that *L* = 2*c*, *w* = 2*a* and 2*b* = 30 μm, the thickness of the ribbon of Metglas^®^ 2826MB3. Obtained values of *N_f_* appear in Table 3.

These *N_f_* values have been obtained assuming χ = 0, but the error generated by Chen et al’s. [25] calculations when assuming $\chi =10^{9}$ is within 1%. From Table 3 it is clear that despite the almost constant value of *R* = 6, the fluxmetric demagnetizing factor changes by almost one order of magnitude, from $1.42\cdot 10^{-4}$ (for the longest strip) to $1.01\cdot 10^{-3}$ (for the shortest one). Hence, despite the strong influence of the length-to-width ratio *R* on the magnetoelastic properties of Metglas^®^ 2826MB3 strips, the most important parameter is actually the demagnetizing factor of each strip, *N*.

So, from our measurements, we must infer that in order to have magnetoelastic resonant platforms showing almost their intrinsic or true *ΔE* effect, we need ribbons with values in the 15 < *R* < 20 range. For such high *R* values the measured *ΔE* ≈ 10%, but when *R* = 5 it reduces to only 6%. The other most-affected parameter is the magnetoelastic coupling coefficient, that reaches 0.3 for 12 < *R* < 20, and reduces to 0.2–0.25 for *R* = 5. The length-to-width aspect ratio has much less influence seems on the width of the measured *ΔE* curve, which remains almost constant (480–560 A/m) for values ranging from 7 < *R* < 20, and increases to 640–800 A/m for *R* ≈ 5. That is, the *R* > 5 criterion must be regarded as a lower limit for this parameter in order to obtain good magnetomechanical response in this type of material. It is the demagnetizing factor of each strip, *N*, that actually determines the magnetoelastic response of the strip material. Having rectangular-shaped magnetoelastic strips with sufficiently high *R* values only guarantees almost-constant values of Young’s moduli (*E_min_*, *Es*) and *ΔE* effect, while the best magnetoelastic parameters are only guaranteed for the strips with the lowest demagnetizing factors.

Following the line of reasoning of our work, future research points toward the determination of true magnetoelastic parameters for magnetoelastic strips with high-enough length-to-width aspect ratio *R* values, along with an adequate estimation of the demagnetizing factor *N* of long rectangular prisms, enabling prediction of the reduction in the magnetoelastic properties of low *R* strips—information of interest when investigating magnetic core materials for remote sensing applications.

## 5. Conclusions

The influence of different length and width (2 < *R = L/w* < 20) commercial Metglas^®^ 2826MB3 magnetoelastic strips in the magnetoelastic properties they exhibit has been extensively analyzed. A significant variation in Young’s modulus and the *ΔE* effect with changing geometry has been observed. Saturation Young’s modulus value *E_S_* and *ΔE* effect magnitude change from 159.9 GPa and *ΔE* = 4% (worst magnetoelastic case, *L* = 10 mm, *w* = 5 mm and *R* = 2), to 164.5 GPa and 9.6% (best magnetoelastic case, *L* = 35 mm, *w* = 1.7 mm and *R* = 20.6). In order to obtain the highest values of the *ΔE* effect, *k* and *Q* magnetoelastic parameters, we have observed that a value of *R* > 14 is needed. However, the effective anisotropy field $H_{k}^{*}$ from the minimum of the *E(H)* curve and its width *ΔH*, seems not to be so strongly influenced by the length-to-width ratio parameter, and a value *R* > 7 is enough to reach their lowest value. This is due to the strong influence—even higher than the influence of the *R* parameter—of the demagnetizing factor, *N*. Thus, a rectangular strip with moderate length-to-width ratio, but with a low demagnetizing factor value, can show an acceptable magnetoelastic response.

From our measurements we infer that the reported value of *R* > 5 needed for a good magnetic and magnetoelastic material response predicted in previous studies [8,9] must be regarded as a lower limit for magnetoelastic resonators. The best magnetoelastic performance is achieved only if the strip *R* ratio is high enough and its demagnetizing factor low. So, when using such amorphous alloys in extremely precise length-controlled magnetic field-controlled actuators, our findings on the variation of magnetoelastic properties with the geometry (size) of the actuator material must be taken into account.

## Figures and Tables

**Table 1:** Magnetoelastic characterization values obtained from the resonance/anti-resonance frequency measurements.

*L* (mm)	*w* (mm)	*R = L/w*	*f_r_ (Hz)*	*E_min_*(GPa)	*E_S_*(GPa)	*ΔE* (%)	$H_{k}^{*}\;(A/m)$	*k_max_*	*Q* (*k_max_)*	*ΔH* (A/m)
35	5	7	63,145	154.3	164.5	6.2	582.4	0.25	30.7	622.2
35	3.33	10.6	63,100	154.1	166.9	7.7	536.9	0.27	24.6	505.0
35	2.5	14	62,240	149.9	164.2	8.7	526.5	0.29	21.5	447.5
35	1.66	20.6	61,965	148.6	164.5	9.6	413.2	0.30	19.2	518.5
30	5	6	73,655	154.3	163.6	5.7	619.8	0.23	33.4	682.9
30	3.33	9.1	73,160	152.2	164.5	7.5	572.8	0.26	25.3	514.5
30	2.5	12	73,397	153.2	166.8	8.1	549.6	0.27	23	487.4
30	1.66	17.6	72,400	149.1	164.9	9.6	432.4	0.29	19.4	524.9
25	5	5	88,782	155.7	164.9	5.6	649.4	0.23	34	688.5
25	3.33	7.6	87,645	151.7	163.9	7.5	583.9	0.26	25.3	494.6
25	2.5	10	88,180	153.6	164.3	6.5	542.5	0.23	29.2	662.1
25	1.66	14.7	87,700	151,9	165.1	8	482.6	0.25	23.4	525.7
20	5	4	110,993	155.7	163.6	4.8	789.0	0.2	39.9	814.5
20	3.33	6.1	109,942	152.8	163.5	6.6	675.7	0.23	28.9	583.9
20	2.5	8	109,760	152.3	165.5	8	669.3	0.24	25.3	521.7
20	1.66	11.8	110,060	153.1	165.1	7.3	522.5	0.22	25.9	572.0
15	5	3	148,012	155.7	162.9	4.4	990.8	0.17	44.3	988.4
15	3.33	4.5	153,475	167.5	177.8	5.8	899.1	0.19	32.8	709.2
15	2.5	6	147,500	154.7	164.7	6.1	776.0	0.19	31.2	708.4
15	1.66	8.8	153,050	166.5	178.6	6.8	702.8	0.19	28.1	641.4
10	5	2	220,400	153.5	159.9	4	1418.4	0.14	48.5	1185.5
10	3.33	3	221,295	154.7	162.7	4.9	1286.0	0.14	39.2	895.1
10	2.5	4	222,902	157	164.1	4.4	1152.7	0.12	44.4	1197.4
10	1.66	5.9	221,175	154.6	164.2	5.9	1005.2	0.14	32.5	819.3

**Table 2:** Magnetoelastic characterization values obtained from the resonance/anti-resonance frequency measurements for strips with *R* = 6 equal or close value.

*L* (mm)	*w* (mm)	*R = L/w*	*E_min_*(GPa)	*E_S_*(GPa)	*ΔE* (%)	$H_{k}^{*}\;(A/m)$	*k_max_*	*Q* (*k_max_)*	*ΔH* (A/m)
30	5	6	154.3	163.6	5.7	619.8	0.23	33.4	682.9
20	3.33	6.1	152.8	163.5	6.6	675.7	0.23	28.9	583.9
15	2.5	6	154.7	164.7	6.1	776.2	0.19	31.2	708.4
10	1.66	5.9	154.6	164.2	5.9	1005.2	0.14	32.5	819.3

**Table 3:** Extrapolated demagnetizing factors for the studied strips with *R* = 6 equal or close value.

L = 2c (mm)	w = 2a (mm)	R = c/a	a/b ^1^	c/(ab)^1/2^	N_f_^2^
30	5	6	166.7	77.5	0.00014248
20	3.33	6.1	111	63.3	0.00019609
15	2.5	6	83.3	54.8	0.00022911
10	1.66	5.9	55.3	44.8	0.00101335

^1^ 2*b* = 30 µm, thickness of the ribbon of Metglas^®^ 2826MB3; ^2^ Fluxmetric demagnetizing factor value, extrapolated by using Table II in [25].
